# Utilization of Smoking Cessation Informational, Interactive, and Online Community Resources as Predictors of Abstinence: Cohort Study

**DOI:** 10.2196/jmir.1018

**Published:** 2008-12-20

**Authors:** Lawrence C An, Barbara A Schillo, Jessie E Saul, Ann H Wendling, Colleen M Klatt, Carla J Berg, Jasjit S Ahulwalia, Annette M Kavanaugh, Matthew Christenson, Michael G Luxenberg

**Affiliations:** ^4^Professional Data Analysts IncMinneapolisMNUSA; ^3^North American Quitline ConsortiumPhoenixAZUSA; ^2^Clearway MinnesotaMinneapolisMNUSA; ^1^University of MinnesotaDepartment of Internal MedicineDivision of General MedicineMinneapolisMNUSA

**Keywords:** Internet, behavior change, smoking, smoking cessation, self-help, interactive, social support, virtual communities, cohort study, multivariate logistic regression, path analysis

## Abstract

**Background:**

The association between greater utilization of Web-assisted tobacco interventions and increased abstinence rates is well recognized. However, there is little information on how utilization of specific website features influences quitting.

**Objective:**

To determine the association between utilization of informational, interactive, and online community resources (eg. bulletin boards) and abstinence rates, with the broader objective to identify potential strategies for improving outcomes for Web-assisted tobacco interventions.

**Methods:**

In Spring 2004, a cohort of 607 quitplan.com users consented to participate in an evaluation of quitplan.com, a Minnesota branded version of QuitNet.com. We developed utilization measures for different site features: general information, interactive diagnostic tools and quit planning tools, online expert counseling, passive (ie, reading of bulletin boards) and active (ie, public posting) online community engagement, and one-to-one messaging with other virtual community members. Using bivariate, multivariate, and path analyses, we examined the relationship between utilization of specific site features and 30-day abstinence at 6 months.

**Results:**

The most commonly used resources were the interactive quit planning tools (used by 77% of site users). Other informational resources (ie, quitting guides) were used more commonly (60% of users) than passive (38%) or active (24%) community features. Online community engagement through one-to-one messaging was low (11%) as was use of online counseling (5%). The 30-day abstinence rate among study participants at 6 months was 9.7% (95% Confidence Interval [CI]  7.3% - 12.1%). In the logistic regression model, neither the demographic data (eg, age, gender, education level, employment, or insurance status) nor the smoking-related data (eg, cigarettes per day, time to first morning cigarette, baseline readiness to quit) nor use of smoking cessation medications entered the model as significant predictors of abstinence. Individuals who used the interactive quit planning tools once, two to three times, or four or more times had an odds of abstinence of 0.65 (95% Confidence Interval [CI] 0.22 - 1.94), 1.87 (95% CI 0.77 - 4.56), and 2.35 (95% CI 1.0 - 5.58), respectively. The use of one-to-one messages (reference = none vs 1 or more) entered the final model as potential predictor for abstinence, though the significance of this measure was marginal (OR = 1.91, 95% CI 0.92 - 3.97, P = .083). In the path analysis, an apparent association between active online community engagement and abstinence was accounted for in large part by increased use of interactive quitting tools and one-to-one messaging.

**Conclusions:**

Use of interactive quitting tools, and perhaps one-to-one messaging with other members of the online community, was associated with increased abstinence rates among quitplan.com users. Designs that facilitate use of these features should be considered.

## Introduction

Improving delivery of tobacco treatment services is a national health priority [1,2]. The Internet is a promising channel to reach a large number of smokers [3]. Approximately 70% of US adults report at least occasional use of the Internet with 60% having Internet access in their homes [4]. Recent trends that demonstrate increasing Internet access among diverse groups are particularly encouraging. Searching for health information online is common, and it is estimated that by 2004 over eight million people had searched online for help to stop smoking [5].

There are several ways in which an individual who is considering stopping tobacco use might find assistance on the Internet. Website visitors may find useful information on how to quit smoking. A recent meta-analysis suggests that simply providing general self-help materials results in a modest increase in quit rates (OR = 1.24, 95% CI 1.07 - 1.45) [6]. Provision of tailored feedback further increases quit rates when compared to untailored or general self-help materials (OR = 1.42, 95% CI 1.26 - 1.61) [6]. A series of randomized studies have demonstrated a modest benefit to providing individually tailored self-help information via the Internet [7-10]. In addition to self-help information, the Internet provides an opportunity for people interested in quitting to receive assistance from others [3,11,12]. Assistance may come from trained experts who can provide advice online on how to overcome specific barriers to quitting. Support may also come from peers who are members of an online community organized around the issue of quitting. An early study by Schneider et al found higher rates of short-term abstinence among individuals randomized to receive access to discussion forums [13]. An observational study by Cobb et al found that engagement with the online community was positively associated with successful quitting [12].

A consistent finding in the evaluation of Web-assisted tobacco interventions is the positive association between website utilization and success in quitting. Lenert reported a positive association between the number of online cessation modules completed and short-term abstinence rates [14]. Cobb reported a positive association between time spent at the website and cessation outcomes. Saul reported a strong association between the number of website visits and abstinence rates at 6-month follow-up [15]. In the American Cancer Society QuitLink study, Pike et al randomized smokers to one of five interactive websites [16]. While there was no overall difference between any of the interactive sites versus a static control site, individuals randomized to interactive websites with higher utilization rates were more likely to succeed in quitting. The authors concluded that interactive smoking cessation websites with high utilization levels can increase quit rates among smokers looking online for help in quitting.

Typically, website utilization has been measured simply in terms of number of modules completed, number of visits, or time spent on the site [10,12,14-17]. Very little information is currently available regarding utilization rates for different site features (eg, general information, tailored self-help functions, and various online community features) and how these patterns of use are associated with quitting. This information is critical to identify potential strategies for improving outcomes for Web-assisted tobacco interventions. If individuals benefit primarily from tailored feedback, then sites could be designed (or redesigned) to highlight this feature more prominently. If individuals benefit primarily from active engagement with the online community, then websites could be altered to facilitate this connection.

In this paper, this critical gap in the literature is addressed by examining in detail the association between utilization of specific website features and cessation outcomes. The website under study is quitplan.com, a Minnesota branded version of QuitNet.com. The quitplan.com website offers general information, tailored feedback, and expert counselor services, as well as a large online community.

## Methods

### Setting

ClearWay Minnesota, a non-profit organization created as part of the state’s settlement with the tobacco industry, provides free access to quitplan.com for all Minnesota residents. Since its initial offering in 2003 through July 2008, over 300,000 individuals have visited the site with over 36,000 registering for services making quitplan.com the most widely used of ClearWay Minnesota’s cessation programs [18]. This study was reviewed by the University of Minnesota’s Institutional Review Board and determined to be exempt under federal guidelines 45 CFR 46.101 (b) for existing data.

### quitplan.com Services

Content and programming for quitplan.com is provided by QuitNet.com. The QuitNet service has been described elsewhere in detail [12]. QuitNet incorporates national tobacco treatment guidelines as a model for best practice for online cessation interventions.

The QuitNet website has an “open” design that is intended to give users easy access to all site features. Information on quitting is presented to site users in the form of general information guides, interactive tools that provide tailored feedback, and online support from expert counselors. General information guides address different stages in the quitting process (eg, Making the decision to quit, Getting ready to quit, Quitting, and Staying quit), quit smoking medications, and frequently asked questions (eg, dealing with symptoms of quitting, including symptoms of withdrawal, weight concerns, etc). There are two categories of interactive tools available at quitplan.com. The first set may be considered diagnostic tools and provide smokers with information about their smoking behaviors. These include the Fagerstrom Tolerance Questionnaire [19], a “Why do you smoke?” questionnaire (adapted from National Cancer Institute Materials), and a Stage of Change [20] assessment. A second set of interactive tools assists individuals in planning their quit attempts. This includes tools to assist in setting quit dates, selecting a medication to help them to quit, and keeping track of days of life and dollars saved since quitting smoking. Individual counseling is available from online counselors. Counselors are certified through the Massachusetts Tobacco Treatment Specialist Training Program [21] and manage the “Ask the Expert” forum where site users may post questions.

The quitplan.com website also allows users to connect to the large QuitNet online community of current and former tobacco users. Site users may browse public discussion boards and forums (bulletin boards) to view posts by members of the online community. Site users may interact with other members by making posts to these public forums or by sending private internal email directly to other members. Approximately 2000 messages per day are posted in public forums with thousands more exchanged privately.

### Study Design

 This study recruited new registrants to quitplan.com from February 2 to April 13, 2004. In order to be eligible, registrants had to (1) be Minnesota residents, (2) be aged 18 years of age or older, (3) be registered as a current tobacco users, and (4) have not already reported quitting at the time of registration. Of 1295 quitplan.com registrants during this period, 1006 were eligible for this study and received an invitation to enroll in the study and complete a follow-up survey in 6 months. An offer of US $10 was made for completion of this survey in 6 months. Of the 1006 eligible registrants, 607 (60.3%) consented to participate in this study.

Follow-up consisted of a mixed-mode follow-up survey using an initial online survey followed by a phone survey of online non-respondents. Participants were mailed a pre-notification letter 6 months after program registration and then sent an email inviting them to complete an online evaluation survey. Reminder emails were sent to non-respondents 3 and 7 days after this initial email. Online survey non-respondents were contacted by phone 12 days after the initial email. The response rate to the follow-up survey was 77.6% (n = 471/607) with 39.4% (n = 239) completing the online survey and 38.2% (n = 232) completing the phone survey.

### Measures

Three data sources are used for this study: registration data, detailed site utilization data, and evaluation survey results. Demographic and clinical variables collected during online registration include age, gender, education, insurance status, readiness to quit, cigarettes smoked per day, and time to first morning cigarette.

To record website utilization information, the QuitNet application server uses a metadata-based tracking model to log all interactions between participants and the system into a relational database. The model is similar to the commonly used W3C Resource Description Framework (RDF) data description model [22], in that it uses three pieces of data (subject-predicate-object, called a triple) to describe any occurring event. This model can include basic page view information (“user 123 viewed page xxx”), fine grained information on content (“page view 2132132 included content xyz”), or feature utilization data (“user 123 successfully completed the quit date wizard”). Each triple is stored in a unique row and contains a unique user identifier and session identifier (which form the subject) and a timestamp, which together, when linked to additional tables describing users and their visit information, comprise a complete tracking system. This not only allows for traditional reporting on “page views” but also high-level reporting on feature utilization.

For the purpose of this analysis we created seven unique utilization measures capturing use of quitplan.com’s informational resources and engagement with the online community. Measures 1 - 4 below assess utilization of different informational resources. Measures 5 - 7 assess engagement with the online community. These measures were defined as follows:

1. General Information: the number of times a user viewed any of the general information guides (ie, Quit Guide, Medication Guide, Frequently Asked Questions).
2. Interactive Diagnostic Tools: the number of times the individual used the Fagerstrom Tolerance Questionnaire, What makes you smoke?, or Readiness to Quit questionnaires.
3. Interactive Quit Planning Tools: the number of times the individual used interactive tools to (a) set their quit date, (b) select a quit smoking medication, or (c) track days and dollars saved since quitting.
4. Counselor Services: number of questions submitted to online expert counselors.
5. Passive Community Engagement: the number of times the user viewed or read discussion board, forum, or journal posts by other community members.
6. Active Community Engagement: the number of times the user made a post to a public discussion board, forum, or journal.
7. One-to-One Messaging: the number of messages sent privately to other community members using the website's internal email system.

The primary outcome is self-reported abstinence for the 30 days prior to the 6-month follow-up evaluation. In determining abstinence, all non-respondents are considered to be continuing to smoke. The follow-up survey also assessed use of smoking cessation medications (nicotine patch, nicotine gum, other nicotine replacement therapy, or bupropion/Zyban) since registration.

### Analysis

Consistent with other studies, raw counts for utilization of different site features were highly skewed. These measures were categorized and median values were reported for each utilization measure category. The correlation between different utilization measures was assessed using a Spearman rho correlation matrix to account for use of categorical measures. Bivariate association between utilization measures and abstinence rates was assessed using Pearson’s chi-square statistic. Logistic regression examined the independent effect of each utilization measure. The dependent variable was self-reported 30-day abstinence. The predictor variables were entered in five blocks using a forward step-wise approach. The first block included demographic characteristics; the second block included smoking variables; the third block included stage of change; the fourth block consisted of the use of any stop smoking medication since quitplan.com registration; and the fifth block consisted of the use of the seven categorical utilization measures.

Considerable interest has been focused on the role of the online community in encouraging smoking cessation. To examine the direct and indirect association between engagement with the online community and abstinence outcomes, path analysis was performed comparing results from two models. Model 1 examined the relationship between Active Community Engagement and Abstinence. Model 2 examined the direct and indirect effects of Active Community Engagement after consideration of potential mediators identified in the logistic regression model described above. Bivariate comparisons and logistic regression models were preformed using SPSS 16.0. Path analyses were performed using AMOS 16.0 software from SPSS Inc.

## Results

### Demographic Characteristics

The demographic and smoking related characteristics of participants are shown in Table 1. The average age of study participants was 38.0 (SD 11.9) years. The majority of participants were female and had completed at least some education after high school. A large majority of site users were employed and had health insurance. The majority of participants smoked more than 15 cigarettes per day and nearly one in three reported smoking within 5 minutes of awakening in the morning. At registration, approximately half of participants reported being in the preparation stage of change. The remainder were in the contemplation or precontemplation stages of change.

**Table 1:** Participant characteristics

		N = 607	%
**Age**			
	18 - 24	82	13.5
	25 - 34	177	29.2
	34 - 44	171	28.2
	45 - 54	123	20.3
	55 or older	54	8.9
**Gender**			
	Male	216	35.6
	Female	391	64.4
**Education**^a^			
	High School or less	100	18.0
	Some college	268	48.1
	College graduate	189	33.9
**Employment Status**^a^			
	Unemployed/other	151	25.2
	Employed for wages	449	74.8
**Health Insurance**^a^			
	Uninsured	79	13.5
	Insured	506	86.5
**Cigarettes/day**			
	< 15	168	27.7
	15 - 24	296	48.8
	25+	143	23.6
**Time 1st a.m. Cigarette**			
	Within 5 minutes	180	29.7
	6 - 30 minutes	259	42.7
	31 - 60 minutes	100	16.5
	After 60 minutes	68	11.2
**Readiness to quit**			
	Precontemplation or Contemplation	302	49.8
	Preparation	305	50.2

^a^Sum less than 607 due to item non-response

### Website Utilization

Participants’ utilization of specific website features is shown in Table 2. Utilization of each of these features was significantly correlated with global utilization measures such as total number of visits to the website and total time spent on the site. Correlations of specific utilization scales with total number of site visits ranged from 0.30 (*P* < .001) for use of counselor services to 0.71 (*P* < .001) for use of interactive quit planning tools.

Use of informational resources was more common than passive or active engagement with the online community. The most commonly used resources were the interactive quit planning tools. Nearly 80% of participants used these tools on at least one occasion, and nearly one-third of participants used these quit planning tools more than four times. The next most commonly used informational resources were the general information guides with over half of participants viewing one or more information guides. Use of the interactive diagnostic tools was less common with somewhat less than half of participants using this resource. Counselor services were used only rarely with less than 5% of participants posting one or more questions to the expert-moderated forums.

Passive engagement with the online community (ie, reading discussion board posts) was more common than active engagement (ie, posting messages). Approximately 40% of participants viewed any posts made by other members of the online community. Active engagement with the community was less common with only approximately one in four participants making any public post. One-to-one messaging between members of the online community was similarly rare with only one in ten participants taking advantage of this feature.

A matrix demonstrating the correlation between these seven utilization measures is shown in Table 3. The highest correlation was 0.617 between the passive and active community engagement measures. The next highest correlation was between use of general information features and the interactive quit planning tools (0.522). Correlations were generally low between use of any counselor services and any of the other utilization measures (all correlations < 0.30).

**Table 2:** Website utilization patterns in the 6 months after initial registration

Informational Resources		Median	Range		N	%
**General Information**		# times guides viewed				
	None	0		0	245	40.4
	Low	1		1 - 2	113	18.6
	Med	4		3 - 5	134	22.1
	High	10		6 - 46	115	18.9
**Interactive Diagnostic Tools**		# times used				
	None	0		0	335	55.2
	1	1		1	127	20.9
	2	2		2	61	10.0
	3+	3		3 - 7	84	13.8
**Interactive Quit Planning Tools**		# times used				
	None	0		0	139	22.9
	1	1		1	145	23.9
	2 - 3	2		2 - 3	143	23.6
	4+	6		4 - 63	180	29.7
**Counselor Services**		# questions sent				
	None	0		0	578	95.2
	1 or more	1		1 - 2	29	4.8
**Passive Online Community Engagement**		# of post viewed				
	None	0		0	374	61.6
	Low	2		1 - 5	112	18.5
	High	20		6 - 56	121	19.9
**Active Online Community Engagement**		# of public posts made				
	None	0		0	463	76.3
	Low	1		1 - 2	72	11.9
	High	7		3 - 42	72	11.9
**One-to-One Messaging**		# of private messages sent				
	None	0		0	543	89.5
	1 or more	3		1 - 643	64	10.5

**Table 3:** Correlation matrix for utilization measures (N = 607)^a^

	Interactive Diagnostic Tools	Interactive Quitting Tools	Counselor Services	Passive Online Community Engagement	Active Online Community Engagement	One-to-One Messaging
General Information	.513	.522	.205	.479	.387	.327
Diagnostic Tools		.509	.226	.361	.309	.215
Quitting Tools			.210	.510	.469	.308
Counselor Services				.256	.278	.275
Passive Online Community Engagement					.617	.470
Active Online Community Engagement						.494

^a^Spearman rho coefficients all significant *P* < .001

### Predictors of Abstinence

Counting non-respondents as smokers, the self-reported 30-day abstinence rate among the 607 study participants was 9.7% (n = 59/607, 95% CI 7.3% - 12.1%). The relationship between utilization of specific website features and abstinence rates is shown in Table 4. In these bivariate comparisons, there was a positive association between self-reported 30-day abstinence rates and use of general information resources, interactive quit planning tools, counselor services, active community engagement, and one-to-one messaging. Neither passive community engagement nor use of the interactive diagnostic tools was significantly associated with abstinence.

In the logistic regression model, neither the demographic data (eg, age, gender, education level, employment, or insurance status) nor the smoking-related data (eg, cigarettes per day, time to first morning cigarette, baseline readiness to quit) entered the model as significant predictors of abstinence. Of the 471 survey respondents, 236 (50.1%) reported use of any smoking cessation medications. Use of smoking cessation medications was not associated with 30-day abstinence at follow-up (abstinence 14.0% for medication users vs 11.1% for non-users, *P* = .34). Medication use did not enter into the regression model (was not significant predictor). Of the seven utilization measures, only two were significant and entered the model: Interactive quit planning tools and one-to-one messages. As a group, the indicator variables representing utilization of interactive quit planning tools, but not the diagnostic tools, were significant with a *P*-value of .03. Compared to individuals who made no use of these tools, individuals who used the interactive quit planning tools once, two to three times, or four or more times had an odds of abstinence of 0.65 (95% CI 0.22 - 1.94), 1.87 (95% CI 0.77 - 4.56), and 2.35 (95% CI 1.0 - 5.58), respectively. The use of one-to-one messages (reference = none vs 1 or more) entered the final model though the significance of this measure was marginal (OR = 1.91, 95% CI 0.92 - 3.97, *P* = .083).

**Figure 1:**
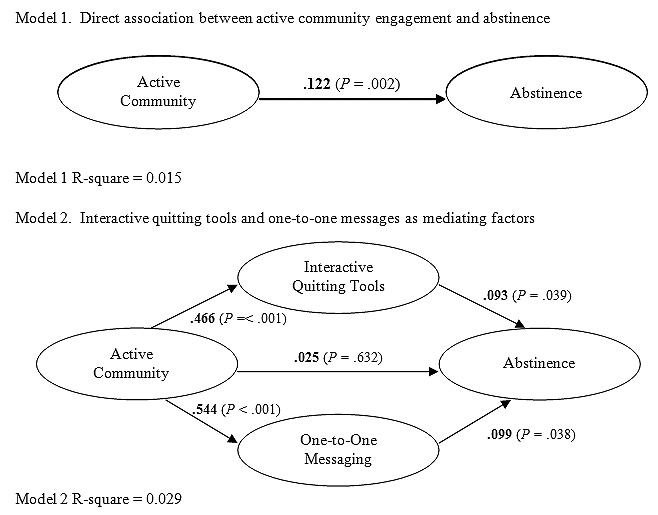
Path analysis of active community engagement and abstinence rates

The results of path analyses are shown in Figure 1. Model 1 is consistent with the results of the bivariate comparison showing a positive association between active community engagement and 30-day abstinence (path coefficient 0.122, *P* < .001). Model 2 explores the direct and indirect effects of active community engagement. Utilization of interactive quit planning tools and one-to-one messaging options were included as potential mediating variables in the path model based upon the findings of the final logistic regression model. In Model 2, active community engagement predicts both use of interactive quitting tools and one-to-one messaging. Each of these variables in turn predicts 30-day abstinence.

An examination of the path coefficients in Model 2 illustrates how the active community path coefficient (0.122) from Model 1 consists of a direct effect on abstinence (0.025) and indirect effects acting through increased use of interactive quit planning tools (.466 x .093 = .043) and one-to-one messaging (.544 x .099 = .054). In fact, these indirect effects for interactive quitting tools (0.043) and one-to-one messaging (0.054) account for a large part (.097/.122 = 79.5%) of the apparent association between active community engagement and abstinence. After accounting for these indirect effects, the direct effect between active community engagement and abstinence is no longer significant.

**Table 4:** Comparison of website feature utilization and 30-day abstinence rates

		30-day Abstinence				*P*-value
		No		Yes		
		N	%	N	%	
**General Information**						.004
	None 0 views	229	93.5	16	6.5	
	Low 1 - 2 views	101	89.4	12	10.6	
	Med 3 - 5 views	124	92.5	10	7.5	
	High 6 - 46 views	94	81.7	21	18.3	
**Interactive Diagnostic Tools**						.266
	None	309	92.2	26	7.8	
	1 use	112	88.2	15	11.8	
	2 uses	52	85.2	9	14.8	
	3+ uses	75	89.3	9	10.7	
**Interactive Quit Planning Tools**						.003
	None	130	93.5	9	6.5	
	1 use	139	95.9	6	4.1	
	2 - 3 uses	127	88.8	16	11.2	
	4+ uses	152	84.4	28	15.6	
**Counselor Services**						.041
	None	525	90.8	53	9.2	
	1 or more use	23	79.3	6	20.7	
**Passive Online Community Engagement**						.198
	None 0 views	342	91.4	32	8.6	
	Low 1 - 5 views	102	91.1	10	8.9	
	High 6 - 56 views	104	86.0	17	14.0	
**Active Online Community Engagement**						.003
	None 0 posts	425	91.8	38	8.2	
	Low 1 - 2 posts	66	91.7	6	8.3	
	High 3 - 42 posts	57	79.2	15	20.8	
**One-to-One Messaging**						.001
	None	498	91.7	45	8.3	
	1 or more	50	78.1	14	21.9	

## Discussion

### Overview

In this observational study of a statewide smoking cessation website, we found an association between the use of interactive quitting tools providing tailored feedback and abstinence rates at 6-month follow-up. Given this finding, it is encouraging to note that nearly 80% of website users made use of one or more of the interactive quit planning tools available through quitplan.com. The finding of positive associations with abstinence related specifically to use of these quit planning tools is consistent with the focus of these tools on key aspects of evidence-based behavioral interventions (ie, setting a quit date, using pharmacological therapy, and follow-up assessment after the quit date) recommended in tobacco treatment guidelines [1].

The abstinence rate observed in this study is consistent with findings from other studies that offered online interactive or tailored feedback. Though timing of the evaluations differ, Etter found that access to a more versus less tailored online program had modest effect on abstinence (10.9% vs 9.8%, *P* = .003) [7]. Swartz et al found that providing tailored online video increased abstinence (12.3% vs 5.0%, *P* < .015) [9]. Pike found a modest benefit to interactive sites with higher versus lower utilization (12.2% vs 10.2%, *P* < .05) [16]. Only Strecher et al reported substantially higher abstinence rates related to an online tailored cessation program (20.1% vs untailored 15.9%, *P* < .001), though in this study all participants had purchased nicotine patches [8]. In total, these findings suggest at most a modest benefit from the use of current smoking cessation websites and substantial room for improvement in the effectiveness of online cessation services.

Interesting findings regarding the potential contribution of different aspects of the online community merit further discussion. Only a minority of participants engaged with the online community in either a passive or active fashion. Interaction with “online experts” was even more rare, a finding that has been reported previously for an online smokeless tobacco intervention [23]. In this study we did not find any association between passive engagement with the online community (ie, reading posts by other members) and abstinence rates. Active engagement with the community at large (through posting to public discussion boards and forums) appeared to be associated with increased abstinence rates in bivariate comparisons. However, logistic regression modeling indicated that active engagement was not an independent predictor of abstinence. Path analyses suggested that the contribution of active community engagement is primarily indirect through an association with increased use of interactive quit planning tools and the one-to-one messaging feature, both of which contribute to greater abstinence rates. These findings suggest a potentially important role for the online community in maintaining user engagement and steering new users to effective resources. Our findings suggest that one-on-one interactions may be needed to provide enough substantive support to assist cessation. Operators of smoking cessation programs might consider website redesign to facilitate these connections.

There was not an independent association between viewing of general information guides and abstinence rates after controlling for utilization of interactive quit planning tools. This is consistent with results of meta-analyses which demonstrate a greater benefit of tailored compared with untailored self-help materials [6]. There was also no independent benefit to site users engaging online with expert counselors. In this case, it may be that use of counselor services was too low to detect a meaningful effect. Future efforts may involve strategies to increase site user engagement with the available expert support functions.

### Limitations

There are several limitations to consider when interpreting the findings presented here. First, it is important to acknowledge this was an observational study and not a randomized controlled trial. We are therefore not able to make causal claims related to the use of different website features and abstinence rates. Selection bias (both in the initial study participation and in website utilization) or unmeasured factors (eg, use of telephone or other counseling services) could have influenced the observed associations. These findings therefore may not generalize to the larger population of smokers who use Web-based cessation services. Second, the findings here are based upon visitors to one (albeit high volume) stop smoking website. Differences in website design would be expected to influence utilization. For example, a site might require completion of certain features as part of registration or strongly promote use of website features in a specific order (ie, “tunnel” design). Besides this basic architecture, other design features such as level of interactivity and incorporation of audio and video might influence utilization. Danaher et al reported much higher utilization for an interactive, “media-rich” website compared to a static text-based comparison site [23]. Thus, our findings on utilization and associations to abstinence for the quitplan.com website may not generalize to other smoking cessation websites, particularly those with substantial differences in design. Third, while this paper advances the knowledge of the potential effects of different types of website features, more fine-grained analyses are still needed. For example, information on the precise timing of the utilization of different website features, and the content and quality of messages exchanged with other members, could further clarify the role of online community engagement in cessation outcomes.

Despite these limitations, the findings reported here contribute to the understanding of effective Web-based tobacco interventions. At present, tailored interventions appear to be a key—and perhaps the key—component to include when creating an effective cessation website. Designers seeking to create effective cessation websites should incorporate interactive assessment and tailored feedback and find ways to feature these resources prominently. Further study of the role that online communities may play in the cessation process is clearly warranted. Future studies could seek to identify and characterize members of online communities who are particularly helpful to others. Eventually interventions may be designed to enhance the quality of online interactions (perhaps through the provision of training in evidence-based practices) to maximize the direct and indirect benefits of online communities.

## Abbreviations

RDF: Resource Description Framework
